# The Appeal for Ex-Service Men

**Published:** 1920-08-28

**Authors:** 


### The Appeal for ex-Service Men.

The appeal in behalf of ex-Service men, to which reference was made in our leading columns last week, seems already to have met with an encouraging response. Although there are, unfortunately, distinct signs of an approaching "slump" in British trade, of which one of the clearest is the increasing number of men reported out of employment in different parts of the country, and although August is always the month in which it is most difficult to obtain new employment, the organisations connected with ex-soldiers and ex-sailors record not a diminution but a slight excess in chances offered for getting these men off the unemployment rolls; and there is hope that the full effect of the appeal will be experienced in September.

All the information that we have been able to gather since Earl Haig wrote his trenchant letter to the Press proves the necessity for keeping the appeal persistently before the notice of the public. Many pitiful tales of distress have been forthcoming in the meantime—such stories as one would have believed impossible at the height of the war, when every soldier was a "hero," and when they were all being told that their dangers and sacrifices would at least be rewarded by finding, on their return, a land "fit for heroes to live in." In this connection it is worth while reading the reports of boards of guardians in counties as far apart as Devonshire, Warwickshire, and Derbyshire. At Derby it was reported at the last meeting of the local board that two-thirds of the men in the casual wards were discharged soldiers.

